# Localized Recrystallization of a Lithium-Metal Anode during Fast Stripping in High-Activity Liquid Electrolytes

**DOI:** 10.1021/acsami.2c17379

**Published:** 2023-01-30

**Authors:** Shang Zhu, Zijian Hong, Zeeshan Ahmad, Venkatasubramanian Viswanathan

**Affiliations:** ‡ Department of Mechanical Engineering, Carnegie Mellon University, Pittsburgh, Pennsylvania15213, United States; § Cyrus Tang Center for Sensor Materials and Applications, State Key Laboratory of Silicon Materials, School of Material Science and Engineering, Zhejiang University, Hangzhou, Zhejiang Province310027, China; ¶ Department of Mechanical Engineering, Texas Tech University, Lubbock, Texas79409, United States; ⊥ Pritzker School of Molecular Engineering, University of Chicago, Chicago, Illinois60637, United States

**Keywords:** lithium-metal anode, phase-field model, localized recrystallization, fast stripping, electrolyte nonidealities

## Abstract

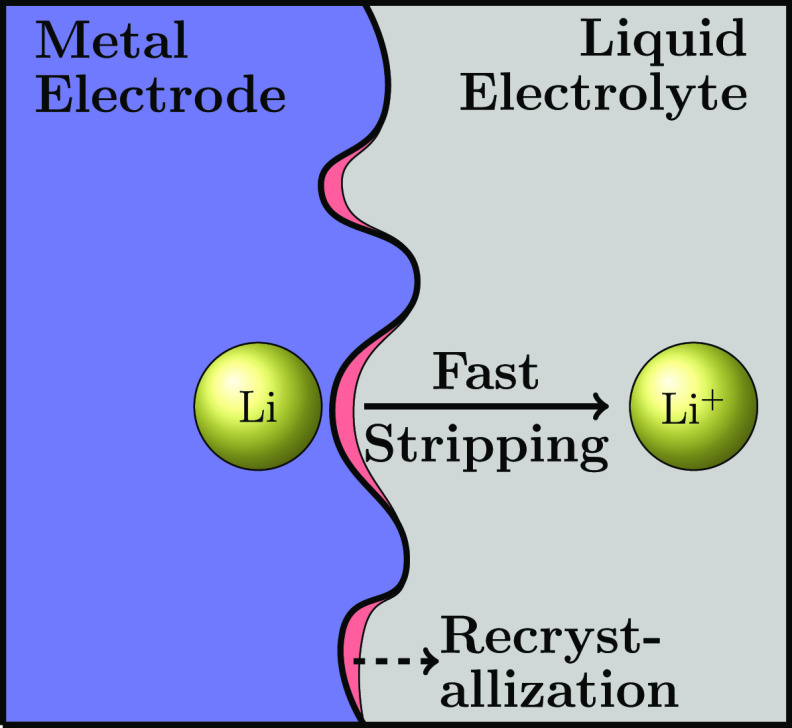

The lithium-metal anode is one of the most promising candidates for “beyond-lithium-ion” batteries thanks to its high specific capacity and low negative electrochemical potential. However, the electrode–electrolyte interface instability hinders its commercialization in rechargeable batteries. During cycles of charging and discharging, the lithium-metal anode is electrochemically plated and stripped along with the morphological evolution, which determines the cycling performance. In this work, with a phase-field model, we computationally characterize the morphological evolution dynamics during the plating and stripping steps at the lithium–metal–electrolyte interface. Our model is valid in a wide range of lithium concentrations in liquid electrolytes by incorporating nonidealities of electrolyte solutions into the interfacial reaction kinetics. Intriguingly, at fast stripping, i.e., high discharging overpotential, we observe an unexpected localized recrystallization phenomenon in high-lithium-ion-concentration valley regions. This recrystallization phenomenon mitigates the overall reaction rate heterogeneity and provides a potential approach to improving the morphological stability. Furthermore, we systematically investigate the correlation between the recrystallization phenomenon and lithium-ion activity and draw a simplified phase diagram for the overpotential-dependent recrystallization.

## 1 Introduction

The lithium-metal anode has attracted a significant amount of research effort due to its high theoretical specific capacity (3860 mAh/g), low negative electrochemical potential (−3.040 V in reference to the standard hydrogen electrode), and low density (0.59 g/cm^3^).^1^ Full cells developed based on a lithium-metal anode could enable high-range electric vehicles, electric trucks, and aircrafts.^2,3^ However, during cycles of charging and discharging lithium-metal anodes, morphological instabilities may occur, associated with widely observed dendrite formation.^4−6^ Furthermore, due to lithium’s high reactivity, dendritic lithium can go through severe side reactions in liquid electrolytes, causing capacity fading and a low Coulombic efficiency of batteries. Lithium dendrites can give rise to even more serious issues such as short-circuiting failures and cell thermal runaways.^7^

Many promising approaches have been proposed to mitigate the morphological instability of a lithium-metal anode.^5,6^ On the electrolyte side, researchers have designed fluorinated solvents,^8−10^ high-concentration electrolytes^11−15^ and localized high-concentration electrolytes,^16,17^ etc. Between the electrolyte and lithium electrode, artificially deposited solid electrolyte interfaces have been proven to be effective in reducing dendrite formation.^18,19^ Both of these methods focus on tuning the ion and charge-transfer kinetics and the mechanical resilience of interfaces so that a more uniform morphology can be enabled. However, theoretical analysis remains underexplored on the ion and charge-transfer kinetics during cycling of the lithium-metal electrode. Although stripping and plating electrochemistry have been widely studied in the research field of electropolishing and electrodeposition,^20−23^ there are limited reports on mechanistic understanding and their implications in the scenario of lithium-metal batteries.^24^ Furthermore, in terms of the lithium electrode, researchers focus extensively on the plating kinetics, which they believe to be the dominant process for dendrite growth.^25,26^ However, it is not until recently that researchers found that lithium stripping kinetics can be critical for the interfacial instabilities associated with void formation.^27−29^

We need to consider a moving boundary when modeling the ion and charge-transfer kinetics during electrochemical plating and stripping steps. The phase-field method, which introduces an order parameter to distinguish between the different phases of lithium (metal or ion), has been the preferred method of choice to examine such a morphological evolution.^30−35^ Recently, advanced phase-field models have been developed to describe lithium plating that incorporates various phenomena such as thermal gradients^36^ and mechanical gradients^37^ near the electrode–electrolyte interfaces. However, to practically simulate a charging and discharging cycle, phase-field models for stripping need to be developed.

In this paper, we have developed a phase-field model to improve the fundamental understanding of the ion and charge-transfer kinetics during cycling of the lithium-metal electrode, i.e. plating and stripping. Our model takes into account the thermodynamic nonidealities, i.e., the lithium-ion activity in electrolyte solutions. We successfully simulate the morphological evolution associated with the plating and stripping steps under varying overpotentials. Interestingly, during fast stripping, high concentrations of lithium ions reverse the direction of interfacial reactions and lead to localized recrystallization. This unexpected phenomenon helps to reduce the heterogeneity of interfacial kinetics and therefore leads to a smooth lithium-metal surface. We rationalize the origin of this phenomenon and find that the high-activity coefficient modifies the exponential terms in the Butler–Volmer reaction equation. This localized recrystallization provides a potential solution for morphological instabilities of lithium-metal batteries by activity modulation.

## 2 Methods

### 2.1 Overview

**Figure 1:**
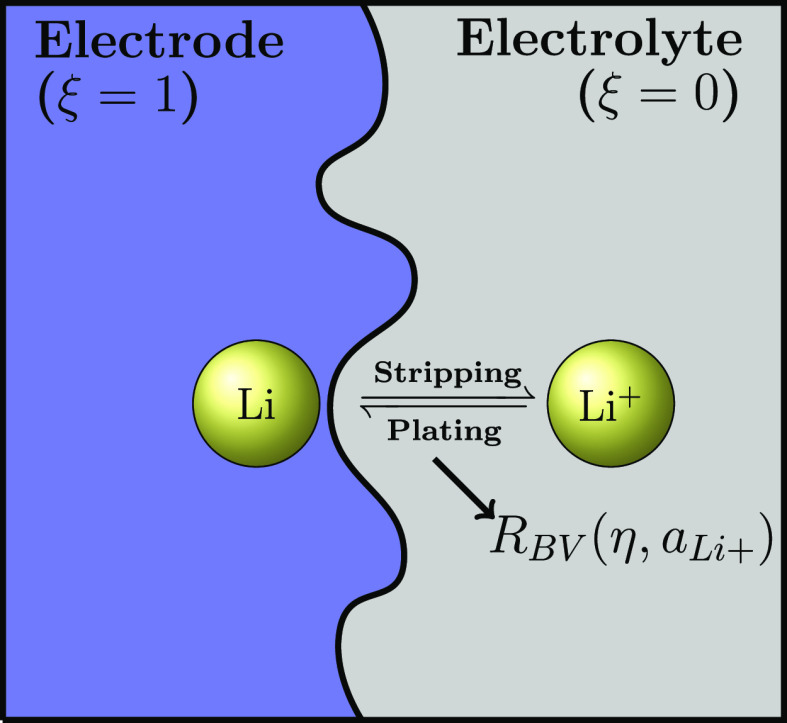
Schematic of the electrodeposition and electrodissolution processes at the lithium electrode–electrolyte interface. Li and Li^+^ are the lithium atom and ion.  is the reaction rate given by the Butler–Volmer kinetics depending on the overpotential η and lithium-ion activity  in liquid electrolytes. The activity term is used to incorporate thermodynamic nonidealities, especially at high concentrations.

We model the electrode–electrolyte interface in two dimensions, with the model schematic shown in Figure 1. In contact with liquid electrolytes, the lithium-metal electrode is plated and stripped during charging and discharging, respectively:
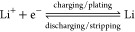
1

Interfacial ion and charge-transfer kinetics are dictated by the electrochemical reaction, ion diffusion, electromigration, and electrical neutrality. We couple these physical relationships and solve them in a phase-field model. In terms of the phase-field equation, we generalize the Allen–Cahn equation with nonlinear reaction kinetics, , which follows the Butler–Volmer fashion, a reasonable approximation of the Marcus–Hush–Chidsey kinetics^38,39^ at moderate overpotentials η. In this work, we highlight the incorporation of lithium-ion activity, , so as to consider the highly concentrated nonideal electrolyte solutions. Mathematical details are provided in the following section.

### 2.2 Governing Equations

We adopt the Butler–Volmer reaction kinetics, , as a function of the overpotential η and lithium-ion activity . Building on our previous work,^26,36,40^ the grand potential functional is used to formulate the phase-field equations, allowing a larger interface thickness for computational convenience while eliminating nonphysical effects.^41,42^ A nonconserved continuous field variable, order parameter ξ ∈ [0, 1], is defined to describe the local phase information, where 1 and 0 correspond to the electrode and electrolyte phases, respectively. The electrode–electrolyte interface is captured by the continuous transition between the electrode and electrolyte phases. Derived from the generalized Allen–Cahn equation, the phase-field equation is given by
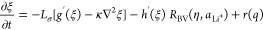
2where the last term *r*(*q*) = *qx* is a Langevin noise in order to incorporate environment fluctuations, where *x* is the random number in the range of [−1, 1] and *q* is the amplitude.^26^ The first term on the right is from the conventional Allen–Cahn equation, which multiplies the interfacial mobility coefficient *L*_σ_ and the free energy gradient over the order parameter. Here the free energy is composed of the bulk energy in a double-well form *g*(ξ) = *b*ξ^2^(1 – ξ)^2^, where *b* is the barrier, and the interfacial energy, which is the product of the interfacial energy coefficient κ and the order parameter gradient. The middle term on the right formulates the reaction rates as the product of the derivative of the interpolation function *h*(ξ) = ξ^3^(6ξ^2^ – 15ξ + 10) and . The interpolation function derivative indicates that redox reactions only happen at interfaces.  is given by
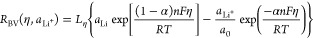
3*L*_η_ is the reaction coefficient, and the two exponential terms correspond to the two opposite reaction directions in eq 1. *a*_Li_ is the activity of solid lithium, which is typically approximated as the lithium mole fraction *c*_Li_. *a*_0_ and *a*_Li^+^_ are the initial and local lithium-ion activities, respectively. α, *n*, *F*, *R*, and *T* represent the charge-transfer coefficient, number of transferred electrons, Faraday constant, ideal gas constant, and applied temperature.

The local lithium-ion activity *a*_Li^+^_ can be written as the product of the activity coefficient and local ion concentration:
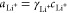
4However, the activity coefficient  is rarely studied in terms of its influence on the interfacial ion and charge-transfer kinetics because most earlier works assume a dilute limit of the electrolyte solutions, where the activity can be approximated by the concentration, i.e., the activity coefficient is set as unity.^25,26^ However, the dilute solution assumption is no longer valid during stripping, especially under a high discharging rate, or in concentrated electrolytes, where the interfacial concentration is rather high. This introduces nonidealities of electrolytes in the interfacial kinetics. Here, the relationship between the activity coefficient  and  is fitted to experimental measurement^43^ and given by
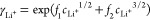
5where *f*_1_ and *f*_2_ are fitted coefficients. It has been reported by Valoen and Reimers that a significant deviation from ideal solutions occurs at high concentrations (>2.5 M).^43^ Experimental measurements have conclusively shown that the activity coefficient can be higher than 1^43−45^ at high concentrations, although a complete microscopic explanation of this phenomenon is lacking. This not only is critical during stripping when the lithium electrode is oxidized into ions, thus building up high concentrations, but it also matters in superconcentrated electrolytes, where the concentrations in the bulk and interface may go as high as 3–4 M.^46^ In terms of the concentrations (mole fractions) of lithium ions  and lithium metal *c*_Li_, we derive them from the chemical potential μ with the following equations:
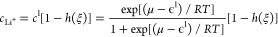
6
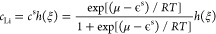
7where *c*^l^ and *c*^s^ are the mole fractions of lithium in the electrolyte and electrode phases, respectively. μ is the chemical potential. ϵ^l^ and ϵ^s^ are the reference chemical potential differences between the lithium species and neutral species in the initial electrolyte and electrode phases, respectively.

Other governing equations include the laws of mass transport and electrical neutrality. The mass transport equation drives the evolution of μ:
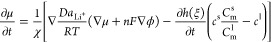
8where *C*_m_^l^ and *C*_m_^s^ are the inverse lithium molar volumes in the electrolyte and electrode phases, respectively. *D* is the diffusion coefficient of lithium ions, depending on the local concentrations, and it is calculated by fitting an empirical relationship, i.e., .^36^*c*_0_ is the initial lithium ion concentration, while *D*_0_ and *k* were obtained by fitting experimental measurements.^43^ Further, χ can be written as^26^
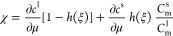
9

With the electrical neutrality assumption, the electric potential ϕ can be characterized by
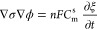
10where σ is the effective conductivity interpolated by the electrode conductivity σ^s^ and the electrolyte conductivity σ^l^, σ = *h*(ξ) σ^s^ + [1 – *h*(ξ)]σ^l^.

In Table 1, we list the physical parameters that we have used in the phase-field simulation. Here, we modified the interfacial mobility, *L*_σ_, from our previous work^26,36^ to improve the algorithm convergence. The electrolyte properties are based on solutions of 1 M LiPF_6_ dissolved in ethylene carbonate (EC) and dimethyl carbonate (DMC) solvents (1:1 volume ratio).^43^ The mole fraction of lithium ion can be approximated with densities of pure solvents. We normalize these physical parameters to further improve the model convergence, where the normalization constants are 1 μm, 1 s, and 2.5 × 10 ^6^ J/m^3^ for length, time, and energy scales, respectively. The parameters after normalization can be found in the open-sourced code package in the Supporting Information (SI).

**Table 1:** Physical Parameters in the Phase-Field Simulation

symbol	variable name	value
*q*	amplitude of Langevin Noise^26^	0.04
*L*_σ_	interfacial mobility^26^	1 × 10 ^–6^m^3^/(J s)
γ	surface tension^26^	0.556 J/m^2^
δ	interface thickness^26^	1 μ m
*b*	barrier coefficient^26^	12γ/δ
κ	gradient energy coefficient^26^	3γδ/2
*L*_η_	reaction coefficient^36^	0.1/s
*c*_0_	initial lithium-ion concentration (mole fraction)^36^	0.067
*a*_0_	initial lithium-ion activity (unity activity coefficient multiplied by the mole fraction)	0.067
α	electron-transfer coefficient^26^	0.5
*n*	number of electrons transferred	1
*F*	Faraday constant	96485 C/mol
*R*	ideal gas constant	8.314 J/(K · mol)
*T*	temperature	300 K
*f*_1_, *f*_2_	empirical coefficients in the activity coefficient relationship^43^	–3.105, 60.18
ϵ^l^	reference μ difference in the electrolyte phase^26^	6.56 × 10^3^J/mol
ϵ^s^	reference μ difference in the electrode phase^26^	–3.44 × 10^4^J/mol
*C*_m_^s^	inverse lithium molar volume in the electrode^26^	7.64 × 10^4^ mol/m^3^
*C*_m_^l^	inverse lithium molar volume in the electrolyte^26^	1.44 × 10^4^ mol/m^3^
*D*_0_	diffusivity of lithium ions without concentration correction^36^	3.197 × 10 ^–10^m^2^/s
*k*	empirical coefficient in the diffusivity function^36^	0.9
σ^s^	conductivity in the electrode^26^	10^7^S/m
σ^l^	conductivity in the electrolyte^26^	1.19 S/m

### 2.3 Model Implementation

Our phase-field model was solved in the fully open-source framework Multiphysics Object-Oriented Simulation Environment (MOOSE).^47^ The system size was 200 × 200 μm^2^, which could provide sufficient space for electrodeposition with an acceptable computational cost. Meshing was composed of 1 × 1 μm^2^ grids, giving decent convergence results for our system.^26^ The Newton method was chosen as the solver type, with a bdf2 scheme and single matrix preconditioning. A transient simulation of 400 s was carried out to capture both stripping and plating dynamics, with adaptive time stepping up to 0.008 s/step. Periodic boundary conditions were selected in the vertical direction for ξ, μ, and ϕ, while Dirichlet-type boundary conditions were applied horizontally. Dirichlet-type values were as follows: ξ = 1/0, μ = 0/0, and ϕ = η_bc_/0 on the left and right ends, respectively, where η_bc_ is the applied electric overpotential at boundaries. In this way, we can tune the overpotentials to investigate the rate dependence of stripping and plating. A negative overpotential triggers a plating step and vice versa for stripping. We fixed the plating overpotential at η_bc_ = −0.16 V and the stripping overpotential at η_bc_ = 0.10 and 0.12 V for the slow and fast stripping cases, respectively. The average *C* rates can be calculated as 15*C*, 20*C*, and 25*C* for the plating, slow stripping, and fast stripping steps. We set these relatively high reaction rates due to the limitation on the computational cost, and they were consistent with previous phase-field models.^25,26,40^ Every time between plating and stripping, a short relaxation period of applying no overpotential was performed to equilibrate the phase field. More details can be found in the SI and our recent work.^48^

## 3 Results and Discussion

### 3.1 Morphological Evolution during Cycling

With the introduced phase-field framework, we simulate the morphological evolution in both the plating and stripping steps. As we mentioned, most previous efforts have focused on the plating step, and a nontrivial extension to model the stripping process is the incorporation of nonidealities, especially in the concentrated solution areas. Next, we will discuss the simulated plating and stripping cycles and investigate the phase-change process.

**Figure 2:**
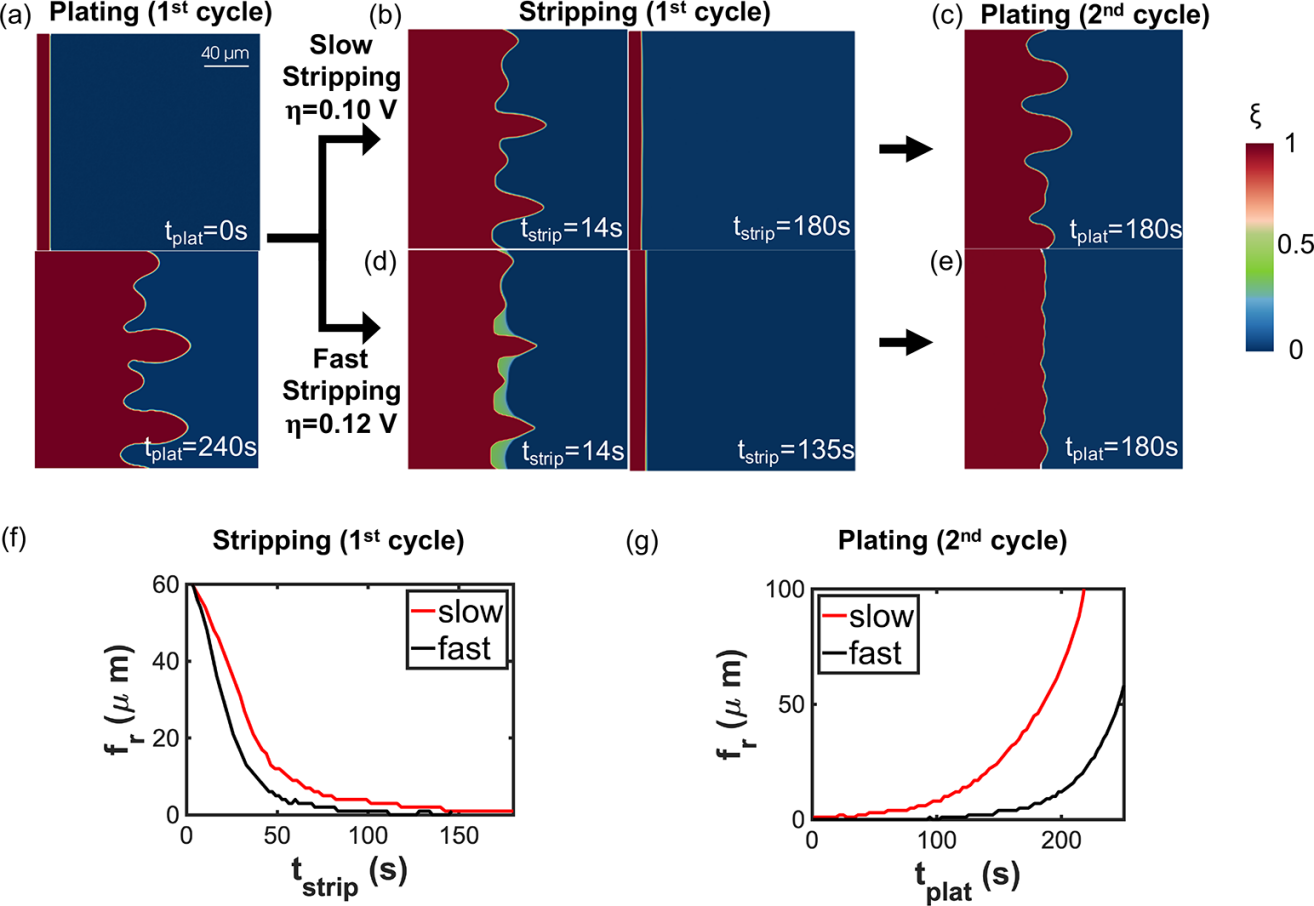
Comparison of the cycling kinetics for slow and fast stripping. (a) Initial and final plating morphology in the first cycle. (b and d) Slow and fast stripping morphology in the first cycle. (c and e) Plating morphology after slow and fast stripping, respectively. (f) Stripping kinetics given by the roughness factor evolution over the stripping time. (g) Plating kinetics given by the roughness factor evolution over the plating time.

In Figure 2, we demonstrate a plating–stripping–plating step (1.5 cycles) starting with a flat electrode surface. The nuclei are initiated due to Langevin noises added in the generalized Allen–Cahn equation. The initial nucleation then grows into larger dendrites at the bottom of Figure 2a. As demonstrated earlier,^26^ we observe dendrite growth due to concentration and electric-field polarization, where the lithium ions are enriched at perturbation tips driven by the local electric field. During stripping, tips undergo a higher dissolution rate than valleys due to the alternating concentration polarization near interfaces, where concentrated areas occur at valleys. The lithium dendrites are removed due to this desirable reaction heterogeneity. The right columns in Figure 2b,d show almost flat morphology after stripping. The stripped surface then serves as the initial morphology for the next plating step, simulating a second charging and discharging cycle. The dendrites are formed again after more lithium is deposited to the electrode, as shown in Figure 2c,e. Our previous work^26^ investigated the rate dependence of electrode morphology during plating and concluded that fast plating causes more severe surface perturbations. Herein, we tune the stripping rate and study its influence on cycling morphology. Fixing the plating overpotential at 0.16 V, we vary the stripping overpotential between 0.10 and 0.12 V. In Figure 2b,d, the stripping kinetics are visualized at two selected time points. The higher stripping overpotential enables fast stripping, where it takes ∼25% less time to finish the stripping step. To obtain a quantitative understanding of the cycling dynamics, we introduce a geometrical descriptor, roughness factor *f*_r_.^40^ It is given by *f*_r_ = *x*_max_ – *x*_min_, where *x*_max_ and *x*_min_ are the maximum and minimum horizontal coordinates of the dendrites. Therefore, a flat electrode surface is featured by a zero *f*_r_. To compare the stripping-rate dependence on the cycling dynamics, we report in Figure 2f,g the temporally evolving curves of *f*_r_ for the stripping step and the following plating step in the second cycle. We find that the curve of fast stripping is always below the slow stripping one, including the ending point of the stripping step. This is still the case if we replace the stripping-time axis with the state of the charge or discharging amount (Figure S2). This indicates that a high stripping rate actually establishes a smoother interface, *f*_r_ = 0 μm compared with that of slow stripping, *f*_r_ = 1 μm. The slightly distinct roughness factors make a huge difference in the following plating step, as displayed in Figure 2g. The higher *f*_r_ after slow stripping advances a lot the nucleation and dendrite growth under the same plating overpotential. The quantitative gap further increases over the plating time. We further confirm this with parts c and e of Figure 2, which clearly show that lithium dendrites are much more significant during the cycles of slow stripping. This finding emphasizes the critical role that the stripping rate may play in the morphological evolution process when cycling batteries.

### 3.2 Localized Recrystallization during Fast Stripping

During fast stripping, one unexpected behavior occurs in that a new phase of a nonzero order parameter is formed at the valleys in the left panel of Figure 2d. The new phase exists in the local area previously occupied by the electrolyte phase. We define this phenomenon as localized recrystallization. The electrode surfaces in contact with recrystallization better preserve their initial interface curvature, while the curvature shifts outside the localized recrystallization zone. This indicates that the stripping rate at valleys is further mitigated compared with that in Figure 2b, likely due to the localized recrystallization phenomenon. The localized recrystallization along with the concentration polarization contributes to the formation of a desirably flat electrode–electrolyte interface. This provides a potential strategy to mitigate dendrite growth and realize stable electrodeposition for next-generation batteries.

**Figure 3:**
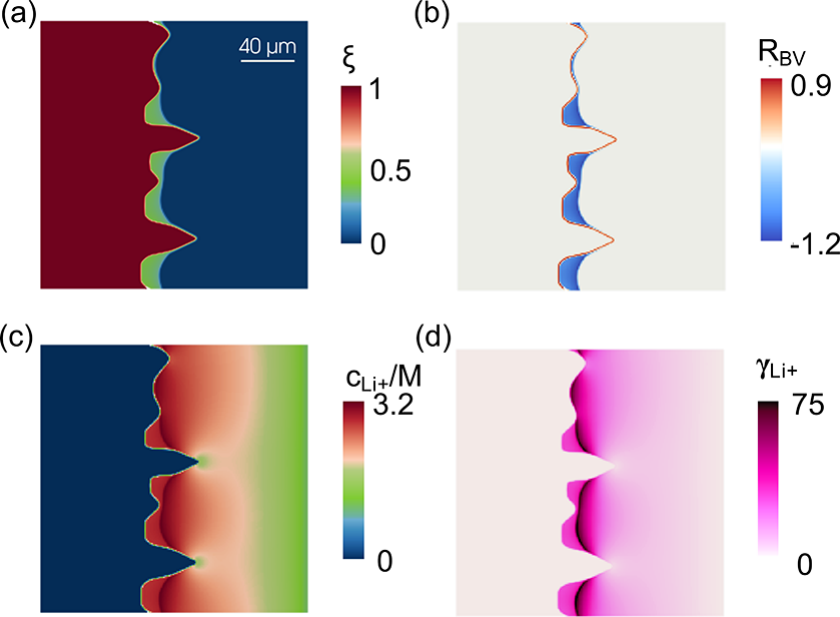
Recrystallization dynamics during fast stripping at *t*_strip_ = 14 s. (a) Order parameter. (b) Butler–Volmer reaction rate, where positive and negative values indicate local stripping and plating, respectively. (c) Lithium-ion concentration. We converted the unit of concentration from mole fraction to moles/liter (M) for analysis convenience. (d) Lithium-ion activity coefficient.

A mechanistic understanding is desired for localized recrystallization during fast stripping. In Figure 3, we visualize the interfacial kinetics of recrystallization from the perspectives of the phase field, reaction rate, lithium-ion concentration, and activity coefficient. The time point is selected at *t*_strip_ = 14 s, consistent with the left panel of Figure 2d. According to Figure 3a, localized recrystallization happens at valleys in contrast to the normal stripping process at tips. Here, the initial morphology is more preserved at valleys in direct contact with the recrystallization field while the triangular surface patterns appear at tips. Figure 3b visualizes the Butler–Volmer reaction rates, indicating that the reaction direction is reversed in the recrystallization areas. This recrystallization phenomenon can well explain the preserved curvature in valleys with the alleviated stripping rate induced by the reverse reaction direction. In Figure 3c, a significant concentration heterogeneity occurs at the electrode–electrolyte interface, induced by the high stripping rate. In the recrystallization region, we identify a local lithium-ion concentration of over 2.5 M. This high concentration area is established at valleys due to its low electric field and relatively uniform concentrations, which prevent the mass transport of lithium ions from valleys to bulk electrolytes through electromigration and diffusion. We visualize the electric potential distribution and electric field in Figure S3. As a result, the localized superconcentration triggers a thermodynamic state highly deviating from ideal solutions, i.e., nonunity lithium-ion activity coefficient in the electrolyte phase. Following experimental measurements from Valoen and Reimers,^43^ we observe that the local activity coefficient can be as high as 30 at valleys, as shown in Figure 3d. One intuitive way to understand the effect of the high activity coefficient is to interpret the activity, the product of the activity coefficient and the physical concentration, as the effective concentration. Although lithium salts may have solubility limits in electrolytes, which are typically less than 5 M in organic solvents,^49^ incorporating a high activity coefficient elevates the effective concentration a lot within the interfacial reaction equation and therefore mitigates and even reverses the stripping rate at valleys. A quantitative analysis can be drawn from the Butler–Volmer reaction kinetics:
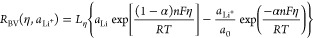
11where *L*_η_ is the reaction coefficient, and the two exponential terms represent two directions of the redox reaction. The reaction rate depends on both the electric overpotential η and local lithium-ion activity . The incorporation of a high activity coefficient may reverse the sign of  if the magnitudes of the two exponential terms are not too off. This only happens at interfaces where the gradient of the interpolation function *h*(ξ) is nonzero because the Butler–Volmer kinetics term is multiplied by *h*′(ξ) in the generalized Allen–Cahn equation. Beyond this area, even higher concentrations occur on the right of the recrystallization field in Figure 3d, but we do not see any reaction happening there. In Figure S4, we include a more quantitative analysis of the four fields introduced in Figure 3. We also visualize the slow stripping kinetics in Figure S5 to compare with the fast stripping case, which indicates smaller concentration and reaction heterogeneity and that moderate activity coefficients trigger no recrystallization.

Surface roughness is another important factor that may influence the recrystallization kinetics of lithium metal.^40^ We investigated its effect on the interfacial kinetics in Figure S6. We varied the roughness factor *f*_r_ by selecting different temporal points during fast stripping. We found that the localized recrystallization phenomenon is significant only when the surface roughness is high, where the concentration and reaction heterogeneity can be built up. Our previous work uncovered the effect of surface roughness on the plating kinetics,^40^ which can be combined with our stripping analysis here and help to form a complete map between the surface roughness and cycling kinetics.

### 3.3 Activity-Modulated Recrystallization and Implications in Lithium-Metal Batteries

**Figure 4:**
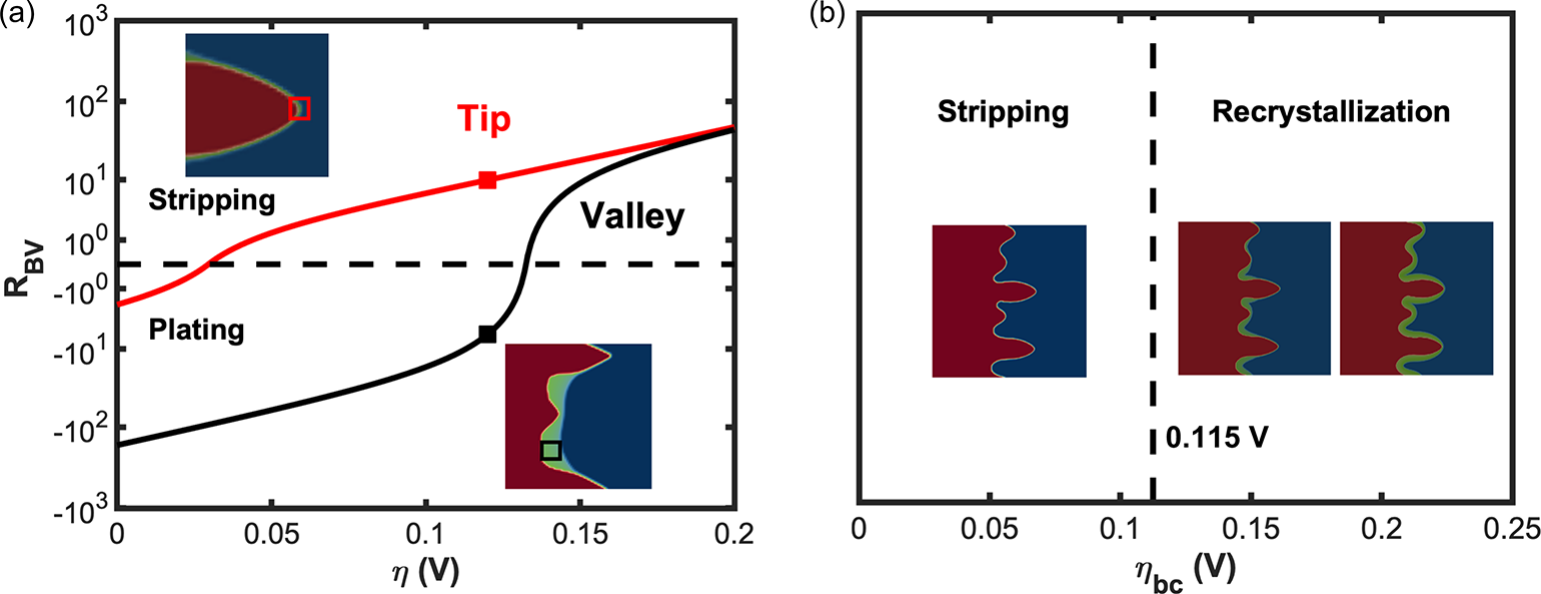
Reaction kinetics and the recrystallization dependence on the stripping overpotentials at boundaries. (a) Butler–Volmer reaction rate as a function of the local overpotentials, given the concentrations and activity coefficients of the tip (0.8 M and 1.96) and valley (2.8 M and 30), respectively. (b) Simplified recrystallization phase diagram on the stripping overpotentials at boundaries. The insert figures show the morphology evolution of 0.10, 0.12, and 0.20 V, where no recrystallization, localized recrystallization at valleys, and full recrystallization are shown. Note that the *x* axis in part b is different from that of part a, in which is the local overpotential that we extract from the simulation.

To investigate the activity modulation on the reaction kinetics and localized recrystallization, we plot the curve of the Butler–Volmer reaction rate as a function of the stripping overpotential in Figure 4a. Two curves have been plotted for the valleys and tips, respectively, during fast stripping. The solid lithium activity, i.e., its mole fraction, is approximated to be 0.5 assuming that *c*^l^ = 1 and *h*(ξ) = 0.5. At the valley, the activity coefficient of ∼30 is triggered by concentrated electrolytes. The concentration we assign to the valley curve is 2.8 M, according to Figure S4. As the prefactor of one exponential term, the high activity coefficient and concentration shift the curve to the right of the axis and the equilibrium overpotential (*R*_BV_ = 0) is approximately 0.13 V. However, the applied electric overpotential is only 0.12 V, which is less than the equilibrium overpotential, as shown by the black dot on the valley curve. This causes the valley reaction rate to be negative, i.e., localized plating and recrystallization. The activity coefficient and concentration decrease to 1.96 and 0.8 M at the tips. The tip curve is to the left of the valley curve, and the equilibrium overpotential here is less than 0.05 V. As a result, the applied overpotential keeps the positive reaction rates, i.e., stripping, at the tips, denoted by the red dot on the curve. In this study, the bulk electrolyte system is based on 1 M LiPF_6_ dissolved in EC and DMC solutions, which display high activity coefficients at high concentrations.^43^ We believe that this recrystallization phenomenon can be generalized to other electrolyte systems with high lithium-ion activities. However, the activity coefficient measurement is relatively underexplored for battery electrolytes, and the microscopic origin of this high activity is lacking, which we hope to address in future work with the help of molecular simulations.

Further, we run phase-field simulations with a large range of stripping overpotentials between 0.05 and 0.25 V. We detect the occurrence of significant recrystallization and summarize them in Figure 4b. The recrystallization phenomenon starts to appear when the stripping overpotential is greater than or equal to 0.115 V. This is due to the activity modulation to the reaction kinetics. When we further increase the stripping overpotential to 0.20 V, recrystallization may happen in an expanded area including both tips and valleys due to the concentration enrichment, which we define as full recrystallization. Herein we take the stripping overpotential of 0.20 V as an example. A higher stripping rate even triggers concentrated solutions at tips, and thus a higher activity coefficient also occurs there. In fact, full recrystallization may not be desirable because both the tip and valley stripping rates are mitigated, causing additional kinetic barriers at the electrode–electrolyte interface. Another potential scenario at an even higher stripping overpotential is that, when concentrations are approaching the solubility limits, the activity coefficients may arrive at a plateau. Then the prefactor of activity may not be able to reverse the magnitudes of the two exponential terms. All of these aspects emphasize that rational control of the stripping rate is necessary to realize smooth electrodeposition and enable safer lithium-metal batteries.

## 4 Conclusions

In summary, we have developed a phase-field model to investigate the plating and stripping kinetics of lithium electrodes in contact with liquid electrolytes. This phase-field model considers the nonidealities of electrolytes with a nontrivial extension of previous models of electrodeposition. The charging and discharging cycles are simulated and display a strong stripping–overpotential dependence in that fast stripping in our test domain enables smoother morphology. Together with our previous analysis of the plating-rate dependence, the simulation results are consistent with experimental observations on the charging- and discharging-rate-dependent interfacial stability of lithium-metal cells,^50^ where a higher stripping rate helps to improve the cycling capacity retention. During fast stripping, an unexpected localized recrystallization phenomenon occurs at the electrode–electrolyte interfaces, which provides a potential suppression strategy for dendrite growth. We explain this phenomenon by uncovering the underlying interfacial kinetics and activity modulation effects on the Butler–Volmer reaction rates. We finally construct a diagram of stripping-rate-driven recrystallization by varying the overpotential between 0.05 and 0.25 V. Further work is required to better understand the microscopic origin of the high activity coefficient and strong nonidealities. Appropriate control of the electrolyte chemistry and compositions, as well as plating and stripping rates, is critical in order to modulate the activities and tune the electrodeposition patterns for next-generation batteries.

## Supporting Information Available

Details on the *C*-rate conversion and supporting figures (PDF)
Implementation code in MOOSE (ZIP)
