# Supplementary material for: Localized Recrystallization of a Lithium-Metal Anode during Fast Stripping in High-Activity Liquid Electrolytes
Source: ACS Appl Mater Interfaces. 2023 Jan 30;15(5):6639–46. doi: 10.1021/acsami.2c17379 (PMC10041533; doi:10.1021/acsami.2c17379)
Supplement: Supplementary file 1 — am2c17379_si_001.pdf [file am2c17379_si_001.pdf]

Supporting Information: Localized Recrystallization of a Lithium Metal Anode during Fast Stripping in High-Activity Liquid Electrolytes

Shang Zhu,[†,‖] Zijian Hong,[†,‡,‖] Zeeshan Ahmad,[¶,§] and Venkatasubramanian Viswanathan[*,†]

†*Department of Mechanical Engineering, Carnegie Mellon University, Pittsburgh, Pennsylvania 15213, United States*

‡*Cyrus Tang Center for Sensor Materials and Applications, State Key Laboratory of Silicon Materials, School of Material Science and Engineering, Zhejiang University, Hangzhou, Zhejiang Province 310027, China*

¶*Department of Mechanical Engineering, Texas Tech University, Lubbock, Texas 79409, United States*

§*Pritzker School of Molecular Engineering, University of Chicago, Chicago, Illinois 60637, United States*

‖*Equal Contribution*

E-mail: venkvis@cmu.edu

Converting the Stripping Rate to C-rate

We can convert the stripping rate into the C-rate by computing the inverse of charging and discharging time. In the fast stripping case, it takes $\Delta t = 145s \approx 0.04h$ to strip from 103 μm to 10 μm ($\Delta d = 93\mu m$) while, in the slow stripping case, it takes $\Delta t = 186s \approx 0.05h$ to do so. Therefore, the C-rate is the inverse of stripping time, that are 25 C and 20 C, respectively. We can also calculate the plating C-rate as 15 C given the plated thickness $\Delta d = 93\mu m$ over $\Delta t = 243s = 0.0675h$. We set these relatively high stripping rates due to the limitation on computational cost and they are consistent with previous phase-field models.[1–3]

Supporting Figures

Figure S 1 demonstrates a full cycle of plating and stripping, where the applied overpotential is set as -0.16 V, 0 V (relaxation period), and 0.10 V in sequence. During plating, a flat electrode surface undergoes nucleation and dendrite growth while the stripping step removes dendritic patterns. At the stripping overpotential of 0.10 V, no recrystallization is observed.

Following Figure 2 in the main text, Figure S 2 provides quantitative results of stripping kinetics as the roughness factor evolution versus the average electrode thickness (depth of discharge). From this figure, we can find that fast stripping is still below that of slow stripping in this format of stripping kinetics and conclude that fast stripping in fact enables a more uniform electrode surface.

Figure S 3 provides the electric potential distribution and electric field (arrows) that drives the electromigration and interfacial concentration heterogeneity. When we apply a higher stripping overpotential, we found that the electromigration is much more significant at tips than valleys, due to the larger magnitude of the tip electric field, as can be seen in Figure S 3. This electromigration-dominant mass transport mitigates the lithium-ion concentrations at tips while a more significant lithium concentration is established at valleys,

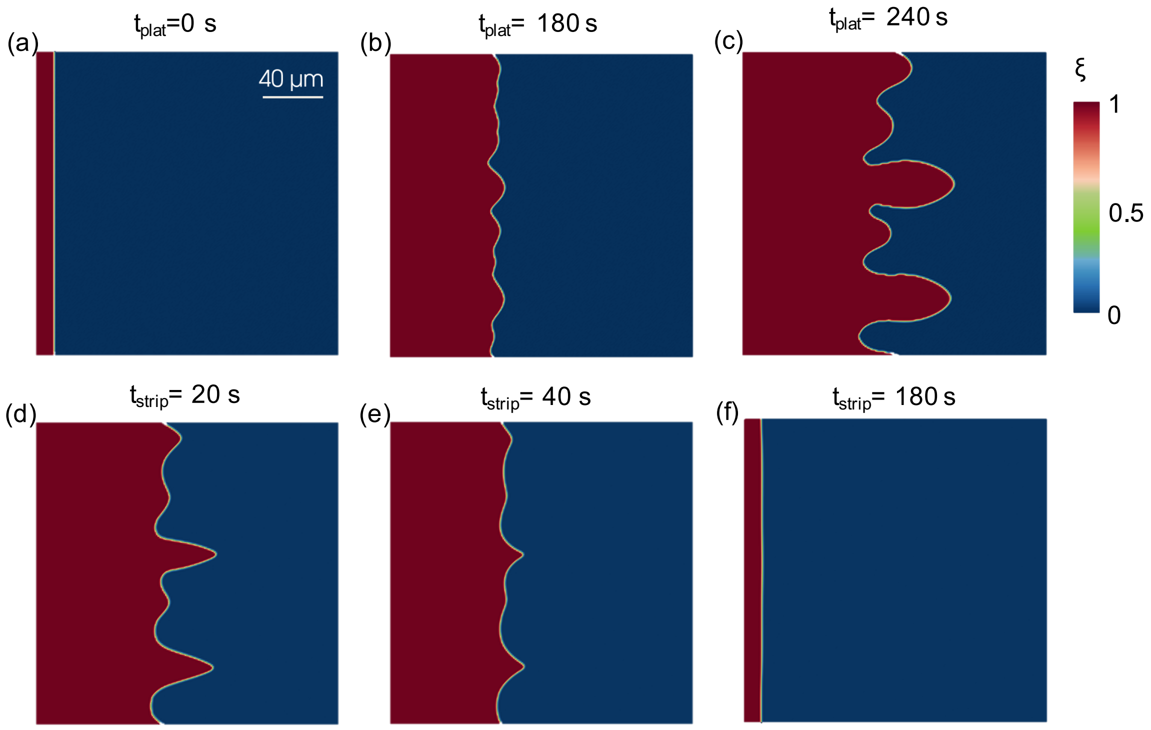

Figure S 1: Lithium plating and stripping in the first cycle. Figures (a)-(c) show the morphological evolution at different plating stages with a plating overpotential of 0.16 V. Figures (d)-(f) show that of the stripping step with a stripping overpotential of 0.10 V.

due to its low electric field and relatively uniform concentrations. This high concentration contributes to high lithium-ion activity and the heterogeneity of equilibrium overpotentials, causing localized recrystallization.

Figure S 4 provides quantitative results of stripping kinetics at valleys and tips, following Figure 3 in the main text. The curves of the order parameter, concentration, ion activity, and reaction rates are given as a function of horizontal coordinate, x_{coord}, by drawing a horizontal line over tips and valleys in the two-dimensional system. From these figures, we can conclude that, approximately, the recrystallization region is of an order parameter of 0.3, a concentration of 2.8 M, an activity coefficient of 30, and a normalized reaction rate of -1.0.

Herein, we also visualize the interfacial dynamics during slow stripping in Figure S 5. With a lower stripping overpotential of 0.10 V, no significant recrystallization has been

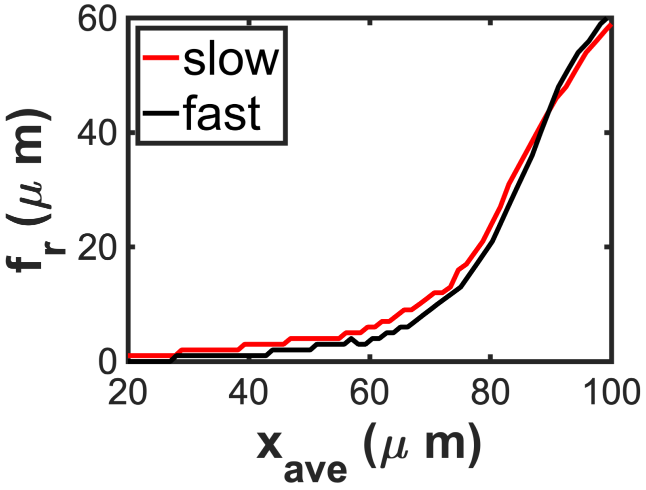

Figure S 2: Stripping kinetics as the roughness factor evolution versus the average electrode thickness.

observed. Therefore, a slightly rougher interface is formed than the fast-stripping case.

Lastly, we investigated the effect of surface roughness on the localized recrystallization phenomenon, as shown in Figure S 6. We selected different temporal points and computed their roughness factors f_r. We found that the recrystallization is significant only when the surface roughness is high, where the concentration and reaction heterogeneity can be built up.

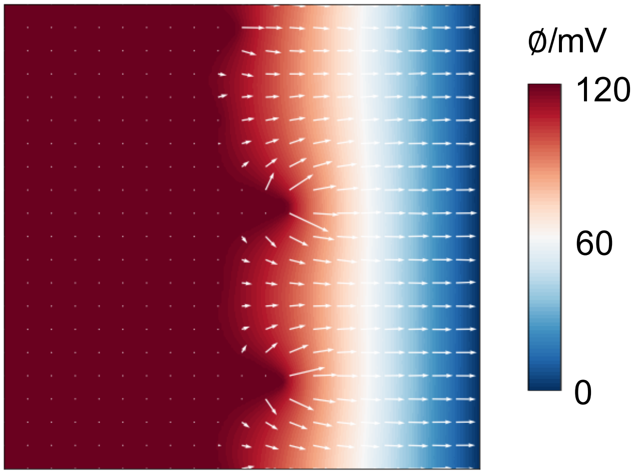

Figure S 3: Electric potential distribution and electric field (arrows) during fast stripping.

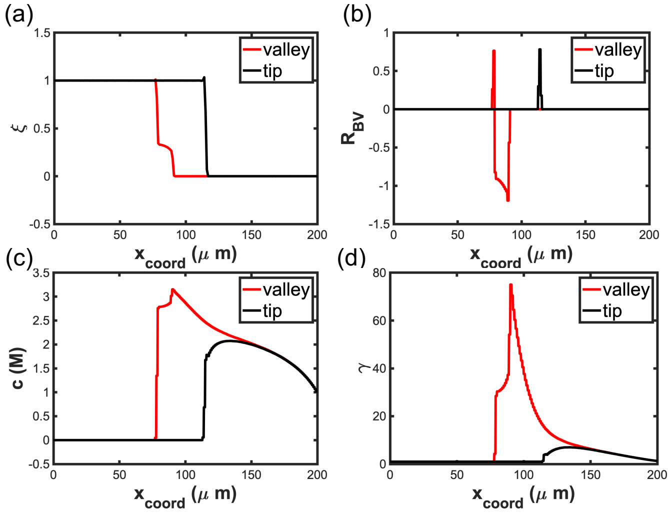

Figure S 4: Quantitative results for the valley and tip kinetics during fast stripping and localized recrystallization. $t_{strip} = 14s$ (a) Order parameter. (b) Butler-Volmer reaction rate, where positive and negative values indicate local stripping and plating, respectively. (c) Lithium-ion concentration. (d) Lithium-ion activity coefficient.

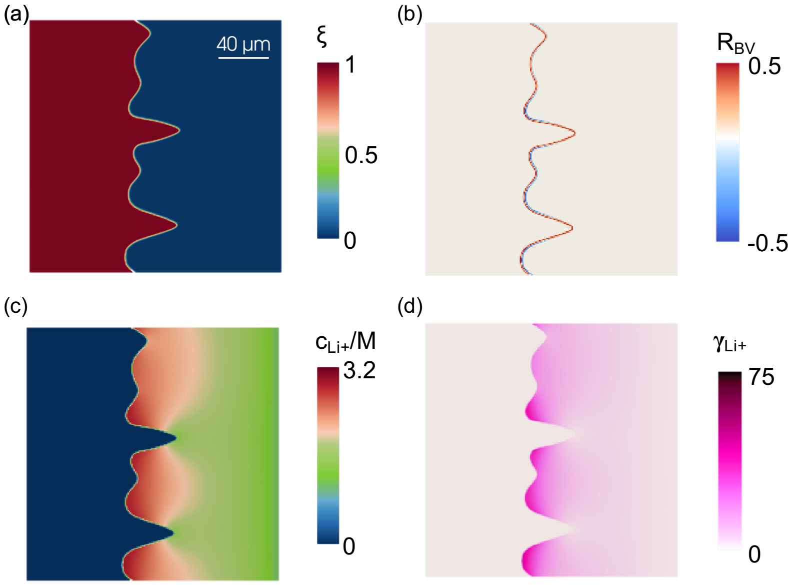

Figure S 5: Interfacial dynamics during slow stripping at $t_{strip} = 14s$. (a) Order parameter. (b) Butler-Volmer reaction rate, where positive and negative values indicate local stripping and plating, respectively. (c) Lithium-ion concentration. (d) Lithium-ion activity coefficient.

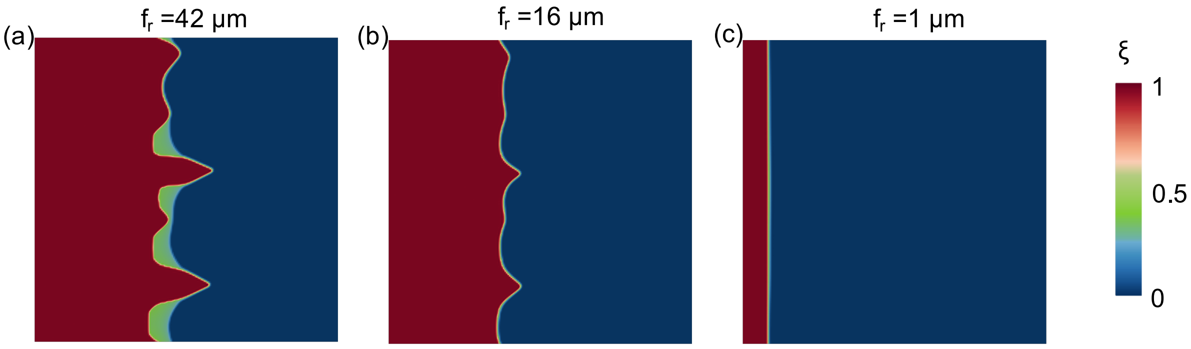

Figure S 6: Effect of surface roughness on the recrystallization phenomenon. The stripping overpotential of 0.12 V. (a) Order parameter distribution at $t_{strip} = 14s$ and $f_r = 42\mu m$. (b) Order parameter distribution at $t_{strip} = 30s$ and $f_r = 16\mu m$. (c) Order parameter distribution at $t_{strip} = 130s$ and $f_r = 1\mu m$.

References

(1) Ahmad, Z.; Hong, Z.; Viswanathan, V. Design rules for liquid crystalline electrolytes for enabling dendrite-free lithium metal batteries. *Proceedings of the National Academy of Sciences* **2020**, *117*, 26672–26680.

(2) Chen, L.; Zhang, H. W.; Liang, L. Y.; Liu, Z.; Qi, Y.; Lu, P.; Chen, J.; Chen, L. Q. Modulation of dendritic patterns during electrodeposition: A nonlinear phase-field model. *Journal of Power Sources* **2015**, *300*, 376–385.

(3) Hong, Z.; Viswanathan, V. Phase-Field Simulations of Lithium Dendrite Growth with Open-Source Software. *ACS Energy Letters* **2018**, *3*, 1737–1743.
